# DPP9 as a Potential Novel Mediator in Gastrointestinal Virus Infection

**DOI:** 10.3390/antiox11112177

**Published:** 2022-11-03

**Authors:** Ángela del Castillo-Izquierdo, José María Moreno-Navarrete, Jessica Latorre, María Arnoriaga-Rodríguez, Marta Ballanti, Giovanni Monteleone, Omero Alessandro Paoluzi, Geltrude Mingrone, Josep Puig, Rafael Ramos, Josep Garre-Olmo, Mariona Jové, Reinald Pamplona, Manuel Portero-Otín, Joaquim Sol, Philippe Lefebvre, Bart Staels, Massimo Federici, José Manuel Fernández-Real, Jordi Mayneris-Perxachs

**Affiliations:** 1 Department of Diabetes, Endocrinology and Nutrition, Dr. Josep Trueta University Hospital, 17007 Girona, Spain; 2 Nutrition, Eumetabolism and Health Group, Girona Biomedical Research Institute (IDIBGI), 17190 Girona, Spain; 3 CIBER Fisiopatología de la Obesidad y Nutrición (CIBERobn), Instituto de Salud Carlos III, 28029 Madrid, Spain; 4 Department of Medical Sciences, School of Medicine, University of Girona, 17004 Girona, Spain; 5 Department of Systems Medicine, University of Rome Tor Vergata, 00133 Rome, Italy; 6 Center for Atherosclerosis, Policlinico Tor Vergata, 00133 Rome, Italy; 7 Department of Internal Medicine, Catholic University, 00168 Rome, Italy; 8 Fondazione Policlinico Universitario A. Gemelli IRCCS, 00168 Rome, Italy; 9 Diabetes and Nutritional Sciences, Hodgkin Building, Guy’s Campus, King’s College London, London WC2R 2LS, UK; 10 Department of Radiology (IDI), Hospital Universitari de Girona Dr. Josep Trueta, 17007 Girona, Spain; 11 Vascular Health Research Group of Girona (ISV-Girona), Institut Universitari d’Investigació en Atenció Primària Jordi Gol (IDIAP Jordi Gol), 08007 Barcelona, Spain; 12 Primary Care Services, Catalan Institute of Health (ICS), 17007 Girona, Spain; 13 Department of Experimental Medicine, University of Lleida-Lleida Biomedical Research Institute (UdL-IRBLleida), 25198 Lleida, Spain; 14 Institut Pasteur de Lille, Université Lille, Inserm, CHU Lille, F-59000 Lille, France

**Keywords:** viral infection, gastrointestinal tract, transcriptomics, metabolomics, SARS-CoV-2

## Abstract

Dipeptidyl peptidase 9 (DPP9) is a member of the dipeptidyl peptidase IV family. Inhibition of DPP9 has recently been shown to activate the nucleotide-binding domain leucine-rich repeat 1 (NLRP1) inflammasome. NLRP1 is known to bind nucleic acids with high affinity and directly interact with double stranded RNA, which plays a key role in viral replication. *DPP9* has also recently emerged as a key gene related to lung-inflammation in critical SARS-CoV-2 infection. Importantly, DPP9 activity is strongly dependent on the oxidative status. Here, we explored the potential role of *DPP9* in the gastrointestinal tract. We performed transcriptomics analyses of colon (microarray, *n* = 37) and jejunal (RNA sequencing, *n* = 31) biopsies from two independent cohorts as well as plasma metabolomics analyses in two independent cohorts (*n* = 37 and *n* = 795). The expression of *DPP9* in the jejunum, colon, and blood was significantly associated with circulating biomarkers of oxidative stress (uric acid, bilirubin). It was also associated positively with the expression of transcription factors (*NRF-2*) and genes (*SOD*, *CAT*, *GPX*) encoding for antioxidant enzymes, but negatively with that of genes (*XDH*, *NOX*) and transcription factors (*NF-KB*) involved in ROS-generating enzymes. Gene co-expression patterns associated with *DPP9* identified several genes participating in antiviral pathways in both tissues. Notably, *DPP9* expression in the colon and plasma was strongly positively associated with several circulating nucleotide catabolites (hypoxanthine, uric acid, 3-ureidopropionic acid) with important roles in the generation of ROS and viral infection, as well as other metabolites related to oxidative stress (Resolvin D1, glutamate-containing dipeptides). Gene-drug enrichment analyses identified artenimol, puromycin, anisomycin, 3-phenyllactic acid, and linezolid as the most promising drugs targeting these *DPP9*-associated genes. We have identified a novel potential pathogenic mechanism of viral infection in the digestive tract and promising existing drugs that can be repositioned against viral infection.

## 1. Introduction

The innate immune system is the host’s first line of defense. It uses a large set of pattern recognition receptors to detect pathogens and injuries [1]. The family of nucleotide-binding domain leucine-rich repeat proteins (NLRPs) constitutes a subset of sensor proteins involved in the initiation of the host innate immune response and mediate innate immunity by forming inflammasomes. NLRP1 was the first inflammasome-forming sensor to be identified [2]. It is highly expressed in epithelial barrier tissues. It has recently been shown that 3C proteases of enteroviruses cleave human NLRP1, leading to its activation by functional degradation [3]. It has also been recently shown that NLRP1 bind nucleic acids with high affinity. Thus, human NLRP1 has been reported to directly interact with double stranded RNA, a typical intermediate of viral replication, predominantly through binging with its leucine-rich repeat, thereby activating the inflammasome [4].

Inflammasome activation may lead to cell death. Therefore, it needs to be tightly regulated. Notably, the dipeptidyl peptidase (DPP) family has shown an important role in immune function [5]. Its enzymatically active members have the rare ability to catalyze the cleavage of Xaa-Pro dipeptides from the N termini of peptides and proteins [6]. The best characterized member of this family is DPP4, which was identified as a functional receptor for MERS-CoV [7]. Notably, circulating DPP4 levels and enzyme activity were associated with viral infections, including hepatitis C virus, Epstein-Barr virus [8] and MERS-CoV [9]. Much less is known about other members such as DPP8 and DPP9 [6]. However, recently, DPP9 has been identified as a novel repressor of the NLRP1 inflammasome activation both in humans [10] and rats [11]. The suppression of NLRP1 by DPP9 requires both its catalytic activity and its binding to NRLP1. Dissociation of the UPA-CARD fragment of NLRP1 from DPP9 results in accumulation of UPA-CARD fragments and subsequent inflammasome activation. DPP9 lacking catalytic activity exerted a stimulatory effect on the NLRP1 inflammasome, which was not observed in a NLRP1-binding-deficiend and catalytic inactive DPP9 [10]. Notably, thiol groups of DPP9 are targets of oxidation-reduction processes that impact upon enzyme activity. Cysteine residues may be responsible for both alkylation- and oxidation-induced inactivation [12]. Interestingly, oxidized DPP9 recovers its catalytic activity when treated with reducing agents such as reduced glutathione (GSH). Remarkably, a recent genome-wide association study (GWAS) in 2244 critically ill SARS-CoV-2 patients from intensive care units in the UK identified DPP9 as a potential previously unrecognized mediator [13]. The *DPP9* locus was also suggested as the peak for severe SARS-CoV-2 in another recent GWAS study of severe SARS-CoV-2 with respiratory failure [14]. In a recent study, transcriptomics analyses from peripheral blood revealed that the expression of *DPP9* was significantly increased in SARS-CoV-2 patients compared to healthy controls or patients with bacterial infections [15].

To date, no study has analysed the expression of *DPP9* in tissues from the digestive system and peripheral leukocytes and the potential relationship with viral infection. Here, we evaluated the transcriptomics profiles of colon (microarray, *n* = 37) and jejunal (RNA sequencing, *n* = 31) samples from two independent cohorts from Italy and Spain, respectively, in relation to the *DPP9* expression. In addition, we also investigated the *DPP9* expression in association with circulating biomarkers of oxidative stress, genes encoding for antioxidant enzymes and transcription factors involved in ROS-generating enzymes. Finally, the plasma metabolic signatures linked to the expression of *DPP9* in the colon (*n* = 31) and plasma (*n* = 795) of a third independent cohort were also evaluated.

## 2. Materials and Methods

### 2.1. Colon Cohort

This cohort comprised human participants recruited as part of the FLOROMIDIA cohort [16], which is a pilot study to investigate OMICS signatures in individuals with a BMI between 20 and 60 kg/m^2^ [16] (Table S1). The colon transcriptomics data from *n* = 37 subjects from the FLOROMIDIA study are deposited in GEO with accession number GSE158237. Inclusion criteria included: Caucasian origin; stable body weight 3 months preceding the study, no systemic disease, and absence of any infections or use of antibiotics one month before the visit. The existence of liver illness, specifically an infection with HBV/HCV and tumor disease, thyroid dysfunction (based on a biochemical work-up), and alcohol consumption (>20 g/day) were all exclusion factors. The Policlinico Tor Vergata University of Rome’s ethical commission (Comitato Etico Indipendente Policlinico Tor Vergata, approval number 113/15, 17 July 2015) validated and accepted the written informed consent provided by each subject.

### 2.2. Jejunum Cohort

This cohort included morbidly obese (BMI > 35 kg/m^2^) patients recruited at the Endocrinology Department of Dr. Josep Trueta University Hospital (*n* = 31) (Table S2) in 2016. All subjects had a stable body weight for at least three months before the study and were of Caucasian origin. Subjects were studied in the post-absorptive state. Exclusion criteria included: (i) any systemic disease other than obesity; (ii) presence of any infections in the previous month before the study; (iii) liver diseases (specifically tumor disease and infections) and thyroid dysfunction, which was specifically excluded by biochemical work-up. The Ethics committee of the Hospital Dr. Josep Trueta revised, validated and approved the protocol (Project Code LBPFGF19, approval number 2016.051). Before being admitted to the study, participants received an explanation of its objective and were asked to sign a written statement of informed consent.

### 2.3. Metabolomics Validation Cohort

To validate the metabolic profiles found in the Colon cohort, we analysed plasma samples provided by *n* = 795 healthy subjects aged > 50 years in fasting conditions from a previous published cohort [17].

### 2.4. Clinical Biochemistry of Colon Cohort

Plasma glucose concentrations were determined twice by the glucose oxidase method using a Beckman glucose analyzer II (Beckman Instruments, Brea, CA, USA). Total plasma cholesterol, HDL-C, and total plasma triglycerides were measured the cholesterol esterase–cholesterol oxidase–peroxidase reaction (Cobas CHOL2, Roche, Basel, Switzerland), the cholesterol esterase–cholesterol oxidase–peroxidase reaction (Cobas HDLC3, Roche, Basel, Switzerland), and the glycerol phosphate oxidase and peroxidase (Cobas TRIGL, Roche, Basel, Switzerland) enzymatic colorimetric methods, respectively.

### 2.5. Clinical Biochemistry of Jejunum Cohort

All participants gave their full consent to the collection of their medical histories and anthropometric data. A sample of blood was given while fasting. Standard laboratory techniques were used to measure the lipid and fasting plasma glucose profiles with an analyzer (CobasR 8000 c702, Roche Diagnostics, Basel, Switzerland). Glycated hemoglobin (HbA1c) was determined by high performance liquid chromatography (ADAMRA1c HA-8180V, ARKRAY, Inc., Kyoto, Japan). Uric acid levels in the jejunum and Imageomics cohorts were measured by standard laboratory methods using an analyzer (CobasR 8000, c702, Roche Diagnostics, Basel, Switzerland).

### 2.6. Colon Transcriptomics

Biopsies from the descendent colon were obtained during routine colonoscopy screening for cancer from patients negative from neoplasms. The biopsies consisted of mucosal samples, which includes epithelium, lamina propria and basal membrane. Samples were maintained in RNAlater (Thermo Fisher Scientific, Waltham, MA, USA), to preserve RNA integrity. Then, samples were immediately transported to the laboratory. The handling of tissue was carried out under strictly aseptic conditions and stored at −80 °C. RNA purification was performed using RNeasy-Tissue Mini-Kit (Qiagen, Hilden, Germany). Total RNA was quantified by Qubit^®^ RNA BR Assay kit (Thermo Fisher Scientific) and the integrity was checked by using the RNA Kit (15NT) on 5300 Fragment Analyzer System (Agilent, Santa Clara, CA, USA). Colon transcriptomics was performed with Affymetrix HUGENE 2.0 ST ARRAY FORMAT 100.

### 2.7. Jejunum Transcriptomics

During gastric by-pass surgery, intestinal epithelium from the jejunum was removed and stored in RNAlater (Thermo Fisher Scientific) to maintain RNA integrity. Following that, samples were sent immediately to the laboratory. Tissue was handled and stored in strict aseptic conditions at a temperature of −80 °C. RNA was purified using the RNeasy-Tissue Mini-Kit (QIAgen). Total RNA was quantified, the integrity was checked and the RNASeq libraries were prepared and sequenced as previously described [18]. RNA-seq reads were mapped against human reference genome (GRCh38) using STAR software version 2.5.3a [19] with ENCODE parameters. Genes were quantified using RSEM version 1.3.0 [20] with default parameters and using the annotation file from GENCODE version 29.

### 2.8. Circulating DPP9 Expression Analysis for Metabolomics Validation Cohort

Blood RNA Kit (Qiagen, Hilden, Germany) was used to isolate RNA from blood. RNA concentrations were assessed with Nanodrop ND-1000 Spectrophotometer (Thermo Fisher Scientific, Waltham, MA, USA). Total RNA was reversed transcribed to cDNA using High Capacity cDNA Archive Kit (Applied Biosystems, Darmstadt, Germany). Gene expression was assessed by real time PCR using an LightCycler^®^ 480 Real-Time PCR System (Roche Diagnostics SL, Barcelona, Spain) with SYBR green technology suitable for relative genetic expression quantification (Roche Diagnostics SL, Barcelona, Spain). Glyceraldehyde-3-phosphate dehydrogenase (GAPDH) was used as endogenous control. The commercially predesigned KiCqStart^®^ primers used were as follows: *DPP9* (forward sequence: 5′-CCACCAAGGTTTATCCAATG-3′, reverse sequence: 5′-ACTCATCGACTTCCTCATAC-3′). The optimal sample concentration was 800 nM.

### 2.9. Plasma Non-Targeted Metabolomics for Colon Cohort

After blood was drawn, the serum was promptly shock-frozen and kept at −80 °C until metabolomics analysis. Samples were thawed prior to the extraction process, and 100 µL of each serum sample was pipetted into randomly selected wells of 96-well plates with a 2 mL capacity. Each 96-well plate received a pipetted sample of human reference plasma. Additionally, samples of human pooled serum were pipetted into six wells. These samples were utilized as technical replicates and for the evaluation of process variability. To act as process blanks, we pipetted 100 µL into 6 wells of each 96-well plate. To assess extraction effectiveness, protein was precipitated, then metabolites were extracted using methanol (475 µL) containing four recovery standard chemicals.

We maintained two aliquots as a reserve and used two for the reverse phase (RP)/Ultra Performance Liquid Chromatography-Tandem Mass Spectrometry (UPLC-MS/MS) analysis in the positive and negative electrospray ionization (ESI) mode. The samples were reconstituted with 50 µL of 0.1% formic acid (FA) for positive ESI mode analysis and 50 µL of 6.5 mM ammonium bicarbonate, pH 8.0, for negative ESI mode analysis. Additionally, QC internal standards were mixed into the reconstitution solvents for both ionization modes in order to monitor instrument performance while also acting as retention reference markers. Thermo Fisher Scientific GmbH, Dreieich, Germany, provided the linear ion trap LTQ XL mass spectrometer and a Waters Acquity UPLC system for the analysis (Waters GmbH, Eschborn, Germany). For acidic (solvent A: 0.1% FA in water, solvent B: 0.1% FA in methanol) and basic (solvent A: 6.5 mM ammonium bicarbonate pH 8.0, B: 6.5 mM ammonium bicarbonate in 95% methanol) mobile phase conditions, optimized for positive and negative ESI mode, respectively, two separate columns (2.1 100 mm Waters BEH C18 1.7 m particle) were used. The columns were produced in a gradient of 99.5% A to 98% B in 11 min at a flow rate of 350 µL/min following injection of the sample extracts. The LTQ XL mass spectrometer’s ESI source was directly coupled to the eluent flow. Turn-by-turn recordings were made of full scan mass spectra (80–1000 *m*/*z*) and data-dependent MS/MS scans with dynamic exclusion. Using Metabolon hardware and software, raw data was retrieved, peaks were detected, and quality control was performed (Durham, NC, USA). Utilizing retention index (RI), accurate mass (+/− 10 ppm), and MS/MS, compounds were identified by comparison to Metabolon’s library entries (Durham, NC, USA). Finally, to verify the consistency of peak identification across the multiple samples, chemicals were manually reviewed and corrected, as appropriate, by data analysts at Metabolon utilizing proprietary visualization and interpretation software. The data was provided with the value at its original scale being the area under the curve of the compound peak. Compounds were screened for defective groups for additional analysis. If a compound was absent from more than half of the samples, it was removed.

### 2.10. Plasma Non-Targeted Metabolomics for Metabolomics Validation Cohort

Ref. [21] in accordance with previously mentioned procedures [20], we extracted metabolites from plasma samples using methanol, which contained phenyl-alanine-C13 as an internal standard. Samples were prepared and the data was collected as previously described [22].

### 2.11. Statistical Analysis

To test normality and homogeneity of variances of clinical variables we used the Shapiro Wilk test. For categorical variables, results are expressed as counts and frequencies; for continuous variables that follow a normal distribution, results are shown as mean and standard deviation (SD); and for non-normally distributed continuous variables, results are shown as interquartile range [IQ]. To determine the correlation between gene transcripts and clinical variables we used Spearman’s rank correlation analysis. False Discovery Rate (FDR) for multiple testing correction.

#### 2.11.1. Gene Co-Expression Patterns

We used Spearman’s rank correlation adjusting *p*-values with the Benjamini-Hochberg correction for multiple testing to identify gene transcripts linked to the *DPP9* expression from colon samples. For jejunal samples, gene transcripts associated with *DPP9* were identified using the DESeq2 R package [23]. Functional pathway enrichment analyses were performed on significant genes based on Reactome Gene sets using Metascape [24] with all measured genes as the gene background. Only terms with *p*-value < 0.01, a minimum count of 2, and an enrichment factor > 1.5 were considered. From these terms, only those with a *q*-value < 0.05 after correcting for multiple testing were considered as significant. *p*-values were calculated based on the cumulative hypergeometric distribution.

#### 2.11.2. Enrichment Networks

As functional enrichment analysis is inherently redundant and thus identifies related or overlapping terms, we employed enrichment networks to collapse redundant terms into single clusters with high intra-cluster similarity. Clusters were obtained from a hierarchical clustering on the enriched pathways. Those pathways with a Kappa scores > 0.3 were considered as a cluster. Only the 10 most significant pathways in each cluster were represented in the networks. The enrichment network was then visualized in using Cytoscape [25]. Each node represents an enriched pathway, where node size is proportional to the number of genes and node colour represents cluster ID.

#### 2.11.3. Gene-Gene Interaction Networks

Gene-gene interaction networks were constructed from significant gene transcripts with the OmniPath, InWeb_IM, and BioGrid databases using Metascape. The resultant network contained the subset of genes that interact with at least one other member of the input list. To simplify large networks and provide more interpretable results, we applied a mature complex identification algorithm (MCODE) [26] to extract densely connected relevant clusters embedded in the large network. Then, a functional enrichment analysis was applied to each cluster independently to annotate its putative biological roles based on the three most significantly enriched Reactome gene sets.

#### 2.11.4. Machine Learning Variable Selection Analysis

We used a machine learning variable selection strategy to identify metabolites associated with *DPP9* levels. After adjusting for age, sex and BMI, we used the random forest-based Boruta algorithm [27]. This is comprised of four steps: randomization, model building, statistical testing and iteration. For statistical testing, we used a Bonferroni corrected two-tailed binomial test to select relevant features and assess whether these features are important (significantly higher, selected), unimportant (significantly lower, rejected) or tentative (non-significant). The algorithm was run with 400 iterations, a 0.005 cut-off for the Bonferroni adjusted *p*-values, and 2000 trees. The associations between *DPP9* expression levels and selected important features were evaluated using a Spearman correlation.

Variable importance measures (VIMs) obtained from random forest models do not provide the sign of the association with the response variable. Therefore, to facilitate the interpretation of the models, the contribution of each metabolites for the prediction of the *DPP9* expression was determined by the exact computation of SHapley Additive exPlanations (SHAP) scores by leveraging the internal structure of the random forests models [28]. The exact computation of SHAP values guarantees that explanations are always consistent and locally accurate. By contrasting the model prediction with and without the feature for each individual, SHAP values establish the significance of a given value in a given feature. As a result, depending on how that characteristic interacts with other aspects of the individual, the same feature with a certain value may have a different SHAP value for different individuals. The R packages “treeshap” and “SHAPforXGBoost” were used to calculate and plot the SHAP scores.

#### 2.11.5. Gene-Drug Enrichment Analyses

Gene-Drug enrichment analyses were performed with DrugBank and STITCH databases using the WebGestalt [29] and GeneSetDB [30], respectively.

## 3. Results

**Figure 1:**
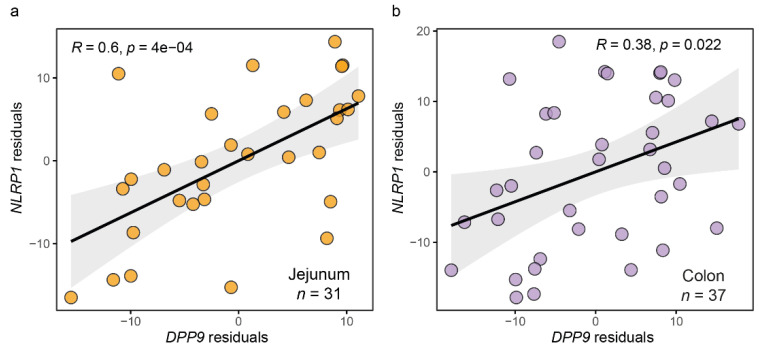
**Associations of *DPP9* expression with the NLRP1 expression.** Scatter plot of the partial Spearman’s rank correlation between DPP9 and NLRP1 expression adjusting for age, sex, and BMI in the (**a**) Jejunum and (**b**) Colon cohorts.

We first assessed the relationship between the expression levels of *DPP9* and *NLRP1* in both the jejunum and colon samples. In both cases, we found a positive association between their expression levels (Figure 1).

### 3.1. The DPP9 Expression Levels in Jejunum, Colon, and Blood Are Strongly Associated with Biomarkers and Genes Involved in Oxidative Stress and Antioxidant Defense

**Figure 2:**
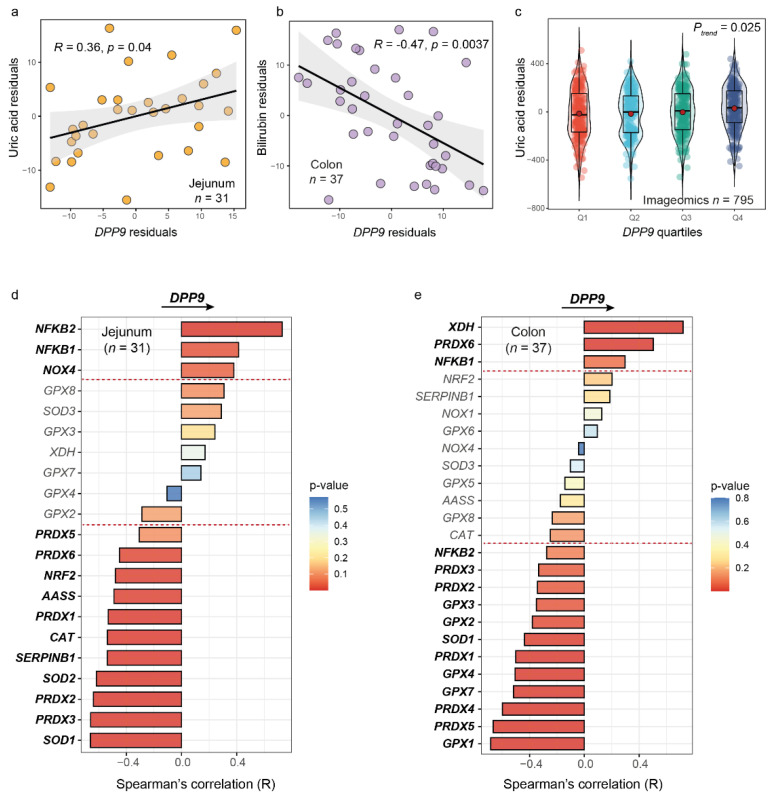
**Associations of biomarkers and genes involved in antioxidant defense and oxidative stress with the expression levels of *DPP9* in jejunum and colon.** Scatter plot of the partial Spearman’s rank correlation (adjusting for age, sex, and BMI) between (**a**) circulating uric acid and the *DPP9* expression in the jejunum, (**b**) plasma bilirubin and the DPP9 expression in the colon. (**c**) Violin plot of the circulating uric acid levels and the quartiles of the *DPP9* expression in blood in the Imageomics cohort (adjusted for age, sex, and BMI). (**d**) Barplot of the Spearman’s correlations of antioxidant and ROS-generating genes with the expression of *DPP9* in the jejunum and (**e**) colon. Bars are coloured according to the *p*-value. Genes above and below the read linies are significant after correcting for multiple comparisons with the Benjamini-Hochberg false discovery rate correction.

As the DPP9 enzymatic activity is strongly dependent on the oxidative status [12], we next evaluated the associations of the expression levels of *DPP9* in jejunum, colon, and blood with biomarkers of oxidative stress and antioxidant status such as uric acid [31,32,33] and bilirubin [34]. After controlling for age, BMI, and sex, we found a significant association between the expression levels of *DPP9* in the jejunum and the circulating uric acid levels (Figure 2a), whereas the colonic *DPP9* expression levels were negatively associated with plasma bilirubin (Figure 2b). In line with this findings, the expression levels of *DPP9* in blood were also positively associated with the circulating uric acid levels in the validation cohort (Figure 2c).

To further assess the effects of the antioxidant status on the *DPP9* expression levels, we evaluated the associations of the jejunal and colonic expression levels of genes coding for important antioxidant enzymes [superoxide dismutase (SOD), glutathione peroxidases (GPXs), peroxiredoxins (PRDXs), and other peroxidases (aminoadipate-semialdehyde synthase (AASS), Catalase (CAT), Serine peptidase inhibitor clade B member 1b (SERPIN1B)] and transcription factors activating genes encoding for antioxidant and detoxyfing enzymes (NRF-2), as well as ROS-generating enzymes [xanthine dehydrogenase/oxidase (XDH/XO), and NADPH oxidase (NOXs)] and transcription factors (NF-kB). Notably, we found strong negative associations among the expression levels of most of these transcription factors and genes encoding for antioxidant enzymes and the *DPP9* expression levels both in the jejunum (Figure 2d) and colon (Figure 2e). Conversely, the expression levels of transcription factors and genes codifying for ROS-generating enzymes were positively associated with the expression levels of *DPP9* in both tissues.

### 3.2. Genes Associated to DPP9 Expression in the Colon Are Involved in Viral Replication, Antiviral Mechanisms and Metabolism of Nucleotides

**Figure 3:**
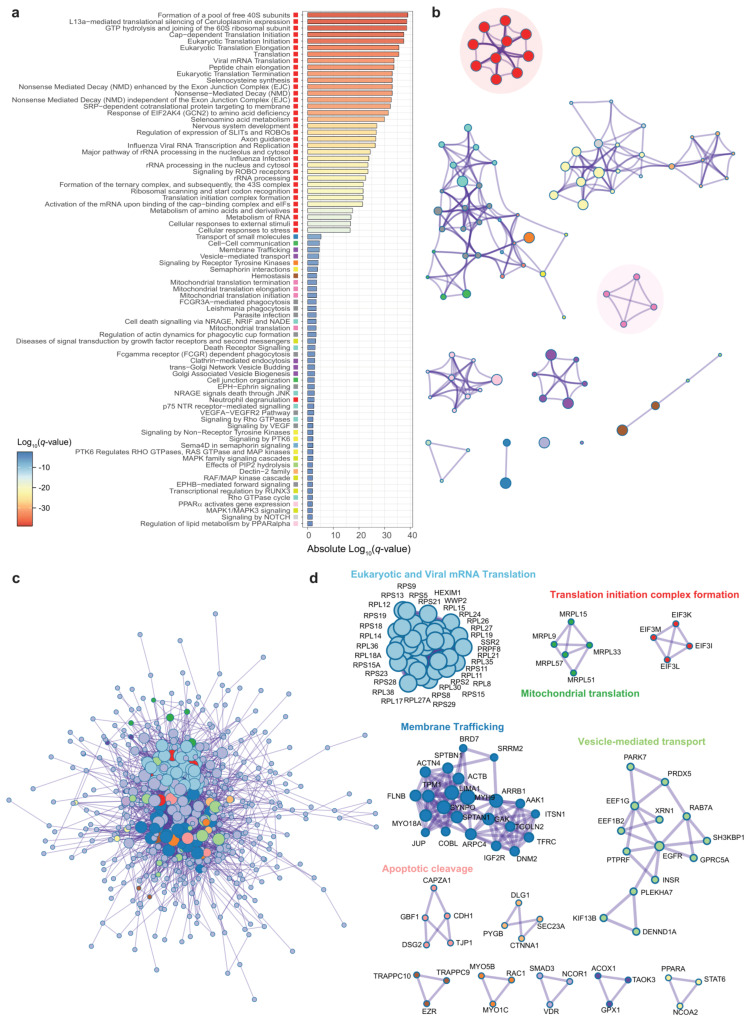
**Pathway enrichment and interactome analysis of gene transcripts significantly associated with *DPP9* in the colon.** (**a**) Bar chart of 80 first enriched Reactome gene sets sorted by Log_10_ (*q*-value). Each gene set have been associated with a cluster represented by different colors. (**b**) Enrichment network. Each node represents a pathway colored by cluster ID. Colors correspond to the clusters showed in panel (**a**). Node size is proportional to the number of input genes falling into that pathway. Edges thickness represents similarity. Only edges with similarity >0.3 are represented. (**c**) Protein-protein interaction network (PPI). (**d**) Clusters of densely connected genes from the PPI obtained using the MCODE algorithm.

We next sought to identify co-expression patterns of genes associated with *DPP9* from the colon microarray. Using Spearman’s rank correlation analyses adjusting *p*-values with the Benjamini-Hochberg correction, we identified 605 out of 21,571 genes significantly associated (P-FDR < 1 *×* 10^−4^) with DPP9, 419 positively and 186 negatively (Table S3). To gain a better insight into the functional pathways involved in these associations we performed an enrichment analysis based on Reactome gene sets using Metascape. Functional enrichment analyses of colonic genes that were significantly associated with the DPP9 expression highlighted an over-representation of 100 pathways (Figure 3a, Table S4).

As functional enrichment analysis is inherently redundant and thus identifies related or overlapping terms, we employed enrichment networks to collapse redundant terms with Kappa similarities >0.3 into single clusters with high intra-cluster similarity. Strikingly, we identified a cluster comprising all terms with the strongest enrichment that included eukaryotic and viral mRNA translation, non-sense mediated mRNA decay (NMD), formation of a pool of free 40S subunits, L13a-mediated translational silencing of ceruloplasmin expression, GTP hydrolysis and joining of the 60S ribosomal subunit, Cap-dependent translation, selenocysteine metabolism and mitochondrial translation (Figure 3b, Table S5). We also identified another cluster containing pathways related to the metabolism of nucleotides (Table S5) that included genes involved in the catabolism of nucleotides such as ENTPD5, GPX1, XDH, GDA, and NT5C2 positively associated with *DPP9* expression.

To gain a better insight and further characterize the relationships among *DPP9*-associated genes we constructed a protein-protein association network from significant gene transcripts with the BioGrid, InWeb_IM, and OmniPath databases (Figure 3c). To simplify the large network and provide more interpretable results, we applied a mature complex identification algorithm (MCODE) to extract densely connected relevant clusters embedded in the large network. Then, a functional enrichment analysis was applied to each cluster independently to annotate its putative biological roles based on the three most significantly enriched Reactome gene sets (Figure 3d, Table S6). From 12 identified significant cluster, three contained genes encoding for thirty-six 40S and 60S ribosomal proteins, five mitochondrial ribosomal proteins (MRPL), and four eukaryotic initiation factor 3s (EIF3s). These clusters were enriched in eukaryotic and viral mRNA translation, mitochondrial translation, and translation initiation complex formation pathways, respectively. All genes from these clusters had negative associations with *DPP9* expression.

### 3.3. Genes Associated to DPP9 Expression in the Jejunum Are Also Involved in Viral Replication and Antiviral Mechanisms

We performed similar analysis from RNA-seq data of jejunal samples. DESeq2 analyses identified 3795 genes significantly associated with *DPP9* (P-FDR < 1 × 10^−4^, Table S7). We thus, limited our gene set functional enrichment analyses to the most significant genes (P-FDR < 1 × 10^−7^). In total, there were 289 pathways over-represented after functional enrichment analyses of jejunal genes significantly associated with the *DPP9* expression (Table S8).

**Figure 4:**
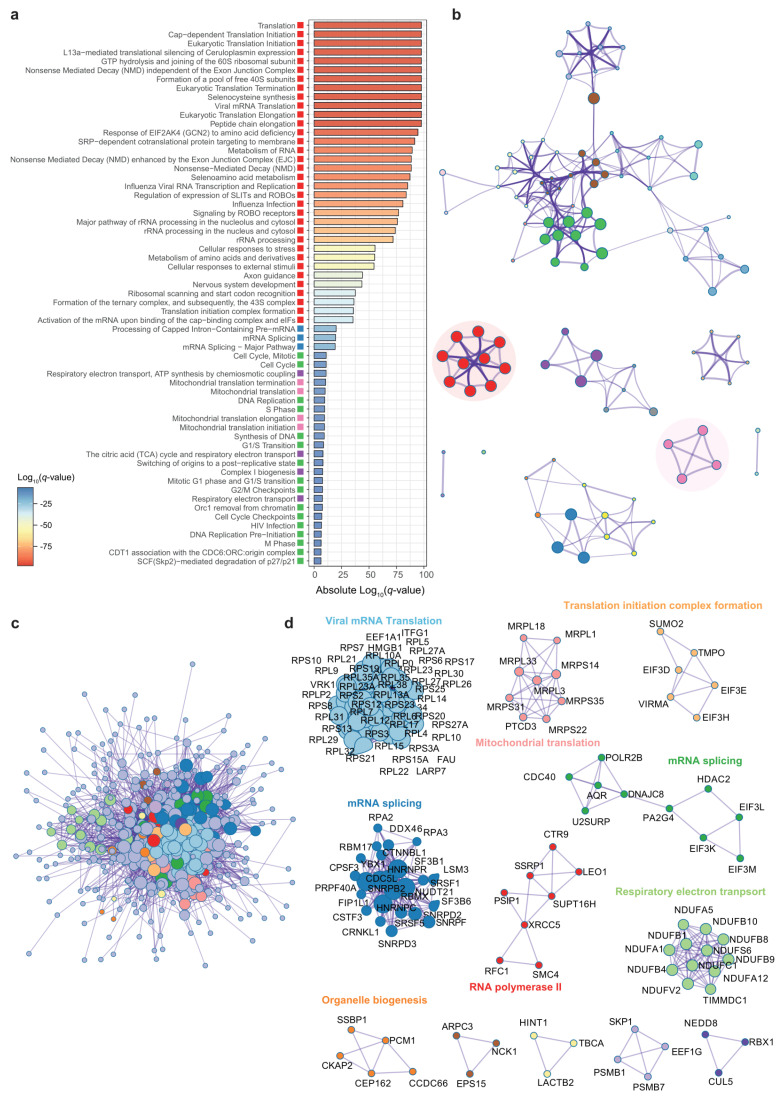
**Pathway enrichment and interactome analysis of gene transcripts significantly associated with *DPP9* in the jejunum.** (**a**) Bar chart of 61 first enriched Reactome gene sets sorted by Log_10_(*q*-value). Each gene set have been associated with a cluster represented by different colors. (**b**) Enrichment network. Each node represents a pathway colored by cluster ID. Colors correspond to the clusters showed in panel (**a**). Node size is proportional to the number of input genes falling into that pathway. Edges thickness represents similarity. Only edges with similarity >0.3 are represented. (**c**) Protein-protein interaction network (PPI). (**d**) Clusters of densely connected genes from the PPI obtained using the MCODE algorithm.

Similarly, to the colon results, we distinguished gene sets negatively associated with *DPP9* expression such as Cap-dependent translation, eukaryotic and viral mRNA translation, L13a-mediated translational silencing of ceruloplasmin expression, GTP hydrolysis and joining of the 60S ribosomal subunit, non-sense mediated mRNA decay, formation of a pool of free 40S subunits, selenocysteine metabolism and mitochondrial translation (Figure 4a, Table S8).

We also identified a cluster in the enrichment network that comprised the most enriched pathways including, once again, eukaryotic and viral mRNA translation, non-sense mediated mRNA decay (NMD), formation of a pool of free 40S subunits, L13a-mediated translational silencing of ceruloplasmin expression, GTP hydrolysis and joining of the 60S ribosomal subunit, Cap-dependent translation, and selenocysteine metabolism (Figure 4b, Table S9). In agreement with the previous results, we also identified three clusters of genes negatively associated with DPP9 in the protein-protein interaction network (Figure 4c). The vast majority of these genes encoded for ribosomal proteins and eIFs and were enriched in eukaryotic and viral mRNA translation, mitochondrial translation, and translation initiation complex formation pathways (Figure 4d, Table S10).

### 3.4. Metabolomics Signatures Associated with DPP9 in Plasma

**Figure 5:**
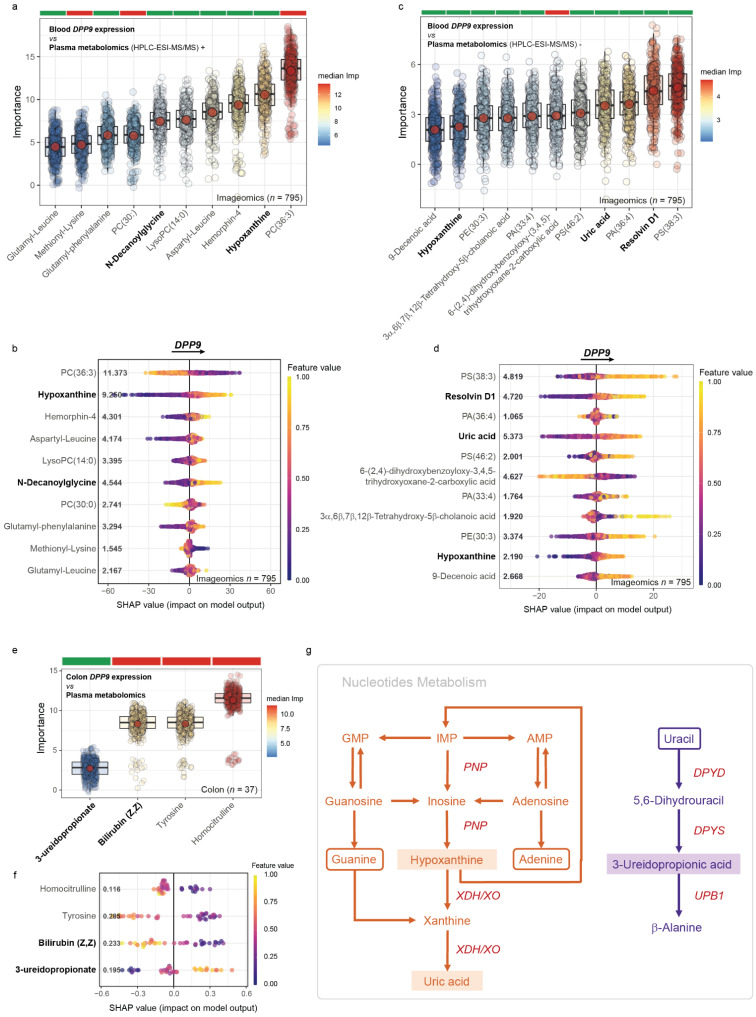
**Metabolomics signatures significantly associated with *DPP9* expression levels in plasma and colon.** (**a**,**b**) Boxplots of the nomalized variable importance measure (VIM) and (**c**,**d**) SHAP summary plots, for the metabolites associated with the blood *DPP9* expression levels in the validation cohort (Imageomics, *n* = 795) measured by HPCL-ESI-MS/MS in positive and negative models, respecitlvely. (**e**) Boxplots of the nomalized variable importance measure and (**f**) SHAP summary plots for the metabolites associated with the *DPP9* expression levels in the colon *(n = 37).* The Boruta results are shown as boxplots of VIM for each selected relevant feature. In the boxplots, the red dot represents the mean and the colour bar above each plot indicates the sign of the correlation among the feature with the *DPP9* expression, with red and green indicating negative and positive correlation, respectively. Significant features were identified using 5000 trees, 500 iterations, and P-Bonferroni < 0.005. In the SHAP summary plot, each dot represents and individual sample. The X-axis represents the SHAP value: the impact of a specific feature (metabolite) on the *DPP9* expression prediction of a specific individual. Features are sorted according to the Boruta results and the overall importance for final prediction (average SHAP values) is shown in bold. Colours represent the values of the metabolites normalized concentrations, ranging from yellow (high concentrations of the specific metabolite) to purple (low concentrations of the specific lipid). (**g**) Outline of purines (orange) and pyrimidines (purple) metabolims from adenylate (AMP), inosylate (IMP) and guanylate (GMP) precursors. Metabolites positively associated with *DPP9* expression are highlighted. Enzymes are shown in red.

We next applied an untargeted metabolomics approach based on HPLC-ESI-MS/MS in both positive and negative modes combined with a random forest -based machine learning variable selection strategy to identify plasma metabolites associated with the expression of *DPP9* in blood in a large-scale validation cohort (*n* = 795). We found that circulating *DPP9* expression levels were associated with several metabolites including nucleotide catabolites related to oxidative stress (hypoxanthine, uric acid), metabolites related to fatty acid oxidation (N-decanoylglycine), lipid mediators involved in inflammatory response and oxidative stress (Resolvin D1), dipeptides (aspartyl-leucine, glutamyl-phenylalanine, glutamyl-leucine, methionyl-lysine), and phospholipids (Figure 5a,b).

To facilitate model interpretation, we computed the SHAP values of the signifcant metabolites (Figure 5c,d). Metabolites related to oxidative stress such as hypoxanthine, uric acid, N-decanoylgylcine, Resolvin D1, and glutamate-containing dipeptides were negatively associated with the blood expression levels of *DPP9* (Figure 5c,d). Notably, metabolites participating in nucleotide metabolism were the ones with strongests impacts on the *DPP9* expression levels. These results were confirmed in the colon discovery cohort, where we found again a positive association with metabolites involved in nucleotide catabolism (3-ureidopropionic acid), but negative associations with metabolites with antioxidant properties (Tyrosine and bilirubin) (Figure 5e,f). A representation of the nucleotides metabolism can be found in Figure 5g.

### 3.5. Promising Drugs Targeting DPP9-Associated Genes

**Figure 6:**
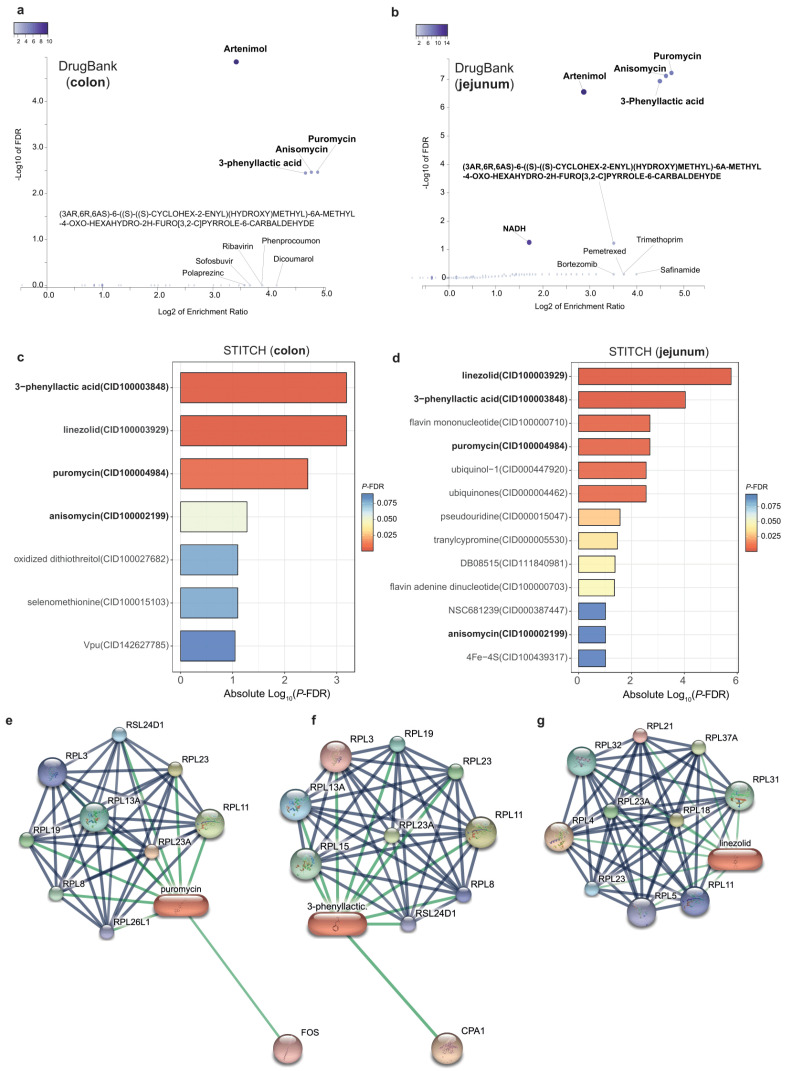
**Gene-drug enrichment analysis of significant genes associated with the expression of *DPP9*.** (**a**) Volcano plot of the gene-drug enrichment results from significant genes associated with *DPP9* in the colon and (**b**) in the jejunum using the DrugBank database. Significant drugs in bold (Spearman’s *p*-value < 0.05, FDR < 0.1). (**c**) Barplot of gene-drug enrichment results from significant genes associated with DPP9 in the colon and (**d**) in the jejunum using the STITCH database. Significant drugs in bold (Spearman’s *p*-value < 0.05, FDR < 0.1). (**e**–**g**) Protein-drug interaction networks of puromycin, 3-phenyllactic acid and linezolid, respectively.

Finally, to identify potential existing drugs targeting genes associate with DPP9, we performed gene-drug enrichment analyses based on both DrugBank and STITCH databases. DrugBank enrichment analyses highlighted artenimol, puromycin, anisomycin, and 3-phenyllactic acid, as the most significant drugs both in colon (Figure 6a) and jejunum samples (Figure 6b), while STITCH-based analyses also identified linezolid (Figure 6c,d). Protein-drug interaction networks of puromycin, 3-phenyllactic acid and linezolid are shown in Figure 6e–g.

## 4. Discussion and Conclusions

DPP9 is a recently identified member of the dipeptidyl peptidase IV enzyme family. Despite being highly prevalent in several tissues, little is known about its biological role compared to the most well-charaterized member of this family, DPP4. However, two recent studies reporting the cryo-electron microscopy structure of human [10] and rat DPP9-NLRP1 [11] revealed DPP9 as a checkpoint for activation of the NLRP1 inflammasome. Inhibition of DPP9 by the small-molecule drug Val-boro-pro results in dissociation of NLRP1 from DPP9 and accumulation of free UPA-CARD fragments that trigger inflammasome activation. Notably, NLRP1 has recently been reported to sense intracellular long double-stranded RNAs, which is implicated in viral cellular replication, which subsequently activates the inflammasome signaling [4]. Notably, recent GWAS studies have identified that *DPP9* was associated with severe COVID-19 [13,14,15]. To gain further insights into the role of *DPP9* in the gastrointestinal tract, here we explored transcriptomic signatures associated with *DPP9* expression in the jejunum and colon.

We performed a functional enrichment analysis to identify the co-expression patterns of genes associated with *DPP9* expression in the colon from microarray data. We found an over-representation of 100 pathways which included terms related to eukaryotic and viral mRNA translation, non-sense mediated mRNA decay (NMD), formation of a pool of free 40S subunits, L13a-mediated translational silencing of ceruloplasmin expression, GTP hydrolysis and joining of the 60S ribosomal subunit, Cap-dependent translation, and selenocysteine metabolism. Another interesting cluster included terms related to mitochondrial translation. Of note, all these pathways have been involved in antiviral mechanisms and contained genes encoding for ribosomal proteins and eukaryotic translation initiation factors (eIFs). Analyses from RNA-seq data of jejunal samples revealed a striking similarity with the colon results. We also identified three clusters of genes negatively associated with *DPP9* in the protein-protein interaction network, most of genes corresponding to ribosomal proteins and eIFs. These genes were also related to eukaryotic and viral mRNA translation, mitochondrial translation, and translation initiation complex formation pathways.

Remarkably, a recent study identifying human proteins that directly and specifically bind to SARS-CoV-2 RNA in infected humans cells highlighted translational initiation, NMD, viral transcription and translation as the cellular pathways most relevant to SARS-CoV-2 infection [35]. In fact, mRNA translation at the endoplasmic reticulum membrane is of well-established importance for coronaviruses [36]. NMD is an eukaryotic RNA quality-control mechanism known to restrict some viruses replication by suppressing and degrading viral RNA within cells [37]. Recently, NMD pathway has shown to interfere with optimal replication of coronaviruses by targeting their RNAs for degradation [38]. Similarly, the cellular ribosomal protein L13a has been identified as an antiviral agent. It is released from the 60S large ribosomal subunit soon after infection and inhibits the translation of a specific viral mRNA [39]. Finally, most mRNAs use the cap-dependent mechanism to initiate translation, where eIFs recognize the cap structure to bind the mRNA to the 40S ribosomal subunit. The non-structural protein 1 (Nsp1), a major virulence factor of SARS-CoVs, has recently been shown to bind to the 40S ribosomal subunit, resulting in a disruption of cap-dependent mRNA translation [40].

We also identified a cluster of pathways involved in nucleotide catabolism positively associated with the expression of *DPP9* in the jejunum. Importantly, virus-infected cells have a metabolic dependency on nucleotide synthesis to support their proliferation [41]. Several signaling pathways such as MYC, RAS, P53 and mTOR are commonly altered in viral infections to increase nucleotide synthesis. Mechanisms used by viruses to increase nucleotide synthesis are not restricted to the induction of anabolic enzymes, but also interference with catabolic pathways [41].

Importantly, the DPP9 activity is highly dependent on the redox state [12]. Hence, DPP9 contains several thiol groups that are targeted by redox processes, thereby impacting on its enzymatic activity. Consistently, we found significant associations among the expression of *DPP9* and markers of oxidative stress such as uric acid and bilirubin. Serum uric acid levels reflect oxidative stress [42]. It is a powerful antioxidant in plasma that can scavenge O_2_^−^ and HO^−^ radicals and has been associated with elevated total serum antioxidant capacity as a mechanism to counteract oxidative damage [33]. Thus, the expression of *DPP9* may be up-regulated to compensate reduced enzymatic activity during oxidative stress. Bilirubin is another important endogenous antioxidant [34]. However, in conditions of oxidative stress, biliverdin reductase (that converts biliverdin to bilirubin) is inactivated, which inhibits the induction of heme oxygenase (that convert heme to biliverdin), thereby inhibiting the formation of bilirubin [43].

In line with these findings, we found strong negatives associations among the expression of genes codifying for antioxidant enzymes (SOD, CAT, GPXs) and the expression of *DPP9* both at the jejunum and colon. SOD, CAT, and glutathione-dependent enzymes such as GPX are the most important antioxidant enzymes. SODs are a family of enzymes that catalyze the dismutation of superoxide into O_2_ and H_2_O_2_, CAT catalyzes the conversion of H_2_O_2_ into H_2_O and O_2_, and GPXs catalyze the conversion of H_2_O_2_ or hydroperoxides to H_2_O and alcohols through the oxidation of GSH to GSSG [44]. The ARE/Nrf-2 pathway is the major player in the induction of the expression of antioxidant genes [45]. Nrf-2 is a transcription factor that is a master regulator of the antioxidant response system by promoting the transcriptional activation of a specific set of target genes containing the antioxidant response elements (AREs) in their promoter regions and encoding for detoxifying and antioxidant enzymes. Notably, the expression of *NRF-2* in the jejunum was strongly negatively associated with the expression of *DPP9.*

Contrary to Nrf-2, NF-kB is involved in the transcription of ROS-generating enzymes [44]. Notably, we found a strong positive association between the expression of *NFKB*s and the expression of *DPP9* in both the jejunum and colon. Among the ROS-generating enzymes, there is a well-established role of XDH and NOX in pathologies related to oxidative stress [46,47]. We found that the *NOX* expression was positively associated with the expression of *DPP9* in the jejunum, whereas the expression of *XDH* had the strongest positive association with *DPP9* in the colon. XDH catalyzes the oxidation of hypoxanthine and xanthine to uric acid in the catabolism of purine nucleotides along with the production of ROS, which represents one of the major sources of free oxygen radicals in human physiology [48]. Serum uric acid levels reflect XDH activity and oxidative stress production [42]. Consistent with these transcriptomic findings, we found positive associations of *DPP9* expression with nucleotide catabolites such as hypoxanthine, uric acid and 3-ureidopropionic acid. Importantly, as mentioned above, the generation of nucleotides is necessary during viral infection to support viral replication [49]. In line with these results, Resolvin D1 was one of the metabolites most strongly associated with the expression of *DPP9* in blood. It is a pro-resolving lipid mediator, mainly derived from docosahexaenoic acid, which has shown to attenuate oxidative stress and apoptosis [50,51]. In fact, resolvins that are formed from DHA, are 100–1000 more potent that DHA itself in protecting against oxidative stress [52]. Several studies provide evidence that serum gamma-glutamyltransferase (GGT) at the physiological range is a sensitive marker of inflammation and oxidative stress [53]. GGT initiates the degradation of extracellular GSH releasing the cysteinyl-glycine dipeptide and transferring the glutamyl part to either water or some amino acids, leading to the production of glutamate-containing dipeptides. Notably, we found several of these dipeptides positively associated with the expression levels of *DPP9.*

Finally, we identified three potential existing drugs targeting genes associate with *DPP9* in both colon and jejunum by gene-drug enrichment analyses. These drugs are artenimol, puromycin, anisomycin, and 3-phenyllactic acid. Notably, a network interaction and molecular docking analysis of 825 differentially expressed genes correlated with ACE2 in SARS-CoV-2 patients highlighted puromycin and anisomycin as the most promising drugs binding protein structures of SARS-CoV-2. The SARS-CoV-2 protein interaction map of Gordon et al. also identified linezolid as a potential antibiotic against SARS-CoV-2 [54]. Molecular docking analyses also revealed artenimol as more potent binders of SARS-CoV-2 spike proteins than hydroxychloroquine [55].

The current study presents some limitations. The jejunum and colon cohorts include mainly subjects with obesity. A higher sample size including subjects without obesity and overweight would be more representative of the general population. Secondly, the nature of our study is cross-sectional. Therefore, we cannot infer causation. Pre-clinical in vitro or in vivo studies up- or down-regulating the expression of *DPP9* followed by infecting cells or rodents with viruses would elucidate the direct role of *DPP9.*

In conclusion, our transcriptomics and metabolomics results highlight a novel potential pathogenic mechanisms of viral infection in the digestive system involving DPP9 which can be targeted by repositioning existing drugs.

## Data Availability

The data presented in this study are available within the article and the Supplementary Material.

## Supplementary Materials

The following supporting information can be downloaded at: https://www.mdpi.com/article/10.3390/antiox11112177/s1, Table S1: Clinical characteristics of the Colon cohort; Table S2: Clinical characteristics of the Jejunum cohort; Table S3: Spearman’s rank correlations between gene transcripts and *DPP9* from the colon transcriptomics; Table S4: Pathway enrichment analysis of the colon genes significantly associated with *DPP9* (P-FDR < 0.0001) using the Reactome gene sets; Table S5: Enrichment networks analysis from colon enrichment analysis using Metascape; Table S6: Clusters and genes identified from the protein-protein interaction network from the *DPP9*-associated colon genes using the MCODE algorithm; Table S7: DESeq2 results for the gene transcripts associated with *DPP9*; Table S8: Pathway enrichment analysis of the jejunum genes significantly and negatively associated with *DPP9* (P-FDR < 1 × 10^−7^) using the Reactome gene sets; Table S9: Enrichment networks analysis from jejunum enrichment analysis using Metascape; Table S10: Clusters and genes identified from the protein-protein interaction network resulting from the *DPP9*-associated jejunal genes using the MCODE algorithm.
